# Using the yes/no recognition response pattern to detect memory malingering

**DOI:** 10.1186/2050-7283-1-12

**Published:** 2013-06-25

**Authors:** Sebastian Schindler, Johanna Kissler, Klaus-Peter Kühl, Rainer Hellweg, Thomas Bengner

**Affiliations:** Abteilung Psychologie, Universität Bielefeld, Bielefeld, Germany; Fachbereich Psychologie, Universität Konstanz, Constance, Germany; Klinik und Hochschulambulanz für Psychiatrie und Psychotherapie, Charité, Campus Benjamin Franklin, Berlin, Germany; Klinik für Psychiatrie und Psychotherapie, Charité, Campus Mitte, Berlin, Germany; Epilepsiezentrum Berlin-Brandenburg, Berlin, Germany

**Keywords:** Assessment, Malingering/symptom validity testing, Learning and memory, Depression, Dementia, Feigning

## Abstract

**Background:**

Detection of feigned neurocognitive deficits is a challenge for neuropsychological assessment. We conducted two studies to examine whether memory malingering is characterized by an elevated proportion of false negatives during yes/no recognition testing and whether this could be a useful measure for assessment.

**Methods:**

Study 1 examined 51 participants claiming compensation due to mental disorders, 51 patients with affective disorders not claiming compensation and 13 patients with established dementia. Claimants were sub-divided into suspected malingerers (n = 11) and non-malingerers (n = 40) according to the Test of Memory Malingering (TOMM). In study 2, non-clinical participants were instructed to either malinger memory deficits due to depression (n = 20), or to perform normally (n = 20).

**Results:**

In study 1, suspected malingerers had more false negative responses on the recognition test than all other groups and false negative responding was correlated with Minnesota-Multiphasic Personality Inventory (MMPI) measures of deception.

In study 2, using a cut-off score derived from the clinical study, the number of false negative responses on the yes/no recognition test predicted group membership with comparable accuracy as the TOMM, combining both measures yielded the best classification. Upon interview, participants suspected the TOMM more often as a malingering test than the yes/no recognition test.

**Conclusion:**

Results indicate that many malingers adopt a strategy of exaggerated false negative responding on a yes/no recognition memory test. This differentiates them from both dementia and affective disorder, recommending false negative responses as an efficient and inconspicuous screening measure of memory malingering.

**Electronic supplementary material:**

The online version of this article (doi:10.1186/2050-7283-1-12) contains supplementary material, which is available to authorized users.

## Background

Malingering is “the intentional production of false or grossly exaggerated physical or psychological symptoms, motivated by external incentives” (American Psychiatric Association 2000, p.739). Malingering of neuropsychological dysfunction frequently occurs in the realm of insurance compensation claims for supposed disability. In fact, a recent study reports abnormal scores on effort tests in up to 44.6% of the investigated claimants (Stevens et al. 2008). Memory disturbances are among the commonly feigned symptoms, even in claimants who do not present with genuine neurological or mental disorders. Therefore, the development of validity assessment methods for complaints about poor memory is vital.

Various strategies have been proposed for the detection of feigned cognitive impairment (for an overview see Rogers 2008). Particularly, forced-choice testing and the floor effect are commonly used detection strategies employed for assessing memory malingering (for an overview see Sweet et al. 2008). Both these methods essentially test for unrealistically poor performance and effort.

The most frequently used forced-choice test using the floor effect for detecting memory malingering is the Test of Memory Malingering (TOMM Tombaugh 1996;Tombaugh 1997;Sharland & Gfeller 2007). The TOMM possesses good specific values (Sollman & Berry 2011), leading to a high positive predictive validity (Vallabhajosula & van Gorp 2001;Batt et al. 2008). Other tests like the Word Memory Test (WMT Green 2003) or the Medical Symptom Validity Test (MSVT Green 2004) have been suggested to exhibit a higher sensitivity than the TOMM, but a recent meta-analysis reported a mean sensitivity of 69% across the five most often examined symptom validity tests including the TOMM, the WMT and the MSVT, with no substantial difference between them (Sollman & Berry 2011).

However, tests that make use of floor effects may be easily recognized as malingering tests by the participant (Sweet et al. 2008;Tan et al. 2002). This will lower their sensitivity to detect malingering. Therefore, it is suggested to rely on multiple indicators of memory malingering to compensate for the moderate sensitivity of each individual symptom validity test (Sollman & Berry 2011). A multi-measure strategy is also recommended by Slick and colleagues’ classic criteria for the detection of neurocognitive malingering (Slick et al. 1999).

Whereas in everyday life, memory problems become most apparent as an inability to voluntarily recall memory contents that is they manifest as free recall failures the veridicality of such free recall failures in suspected malingerers is experimentally practically impossible to assess. There is no way of knowing, whether an examinee is purposefully withholding memory contents or truly experiencing a failure to recollect. The situation changes on recognition tests, where participants have to respond to every single presented item. Recognition tests are normally easier to master than free recall tests, because they do not require voluntary item generation (Haist et al. 1992;see also Gillund & Shiffrin 1984), however, crucially for the present purpose, performance patterns on tests where each item requires a response may also be useful to detect feigned or grossly exaggerated memory deficits.

In the present study, yes/no recognition memory is investigated as one such measure for the detection of memory malingering. In yes/no recognition tasks, participants are presented with only one item at a time, and have to decide if the item has been presented during the study phase or not. While forced-choice recognition, as implemented for instance in the TOMM, allows the distinction of hits and misses only, responses during yes/no recognition tasks can be classified as true positives, true negatives, false positives, and false negatives, affording more precise characterization of response patterns and the application of signal detection measures.

Indeed, earlier studies already noted that qualitatively altered response patterns during the yes/no recognition trial of the California Verbal Learning Test may be useful to detect memory malingering (Trueblood & Schmidt 1993;Millis et al. 1995;Greve et al. 2009a). Claimants classified as probable malingerers have been observed to be characterized by fewer false positive responses than either healthy people or truly memory disturbed patients. The corresponding increase in false negative responses may stem from the fact that individuals feigning or exaggerating memory deficits follow the naive hypothesis that memory disorder is mainly characterized by an almost total failure to encode items presented for learning. That is, lay people may hypothesize that ‘everything will be new all the time’ to people with a genuine memory disorder, leading them to deny having seen any previously presented items. However, empirically this turns out to be untrue. In fact, people with memory disorders are often characterized by a performance suggesting that many things seem vaguely familiar to them, leading them to accept many more items as old and ‘presented before’ than healthy people would, especially in immediate recognition (Bartlett et al. 1989;Fahlander et al. 2002;Stavitsky et al. 2006;Bengner et al. 2006;Huh et al. 2006;Hudon et al. 2009;Werheid et al. 2010;Deason et al. 2012). Although unusual ‘yes/no’ recognition memory patterns in probable malingerers have been noted before, their extent and discriminatory power have never been specifically examined in a targeted study.

The present paper covers two consecutive studies which aim to fill this gap. In a first clinical study, ‘yes/no’ recognition performance was compared between probably malingering and probably non-malingering claimants, inpatients with dementia and inpatients with affective disorder. Dementia was chosen as a clinical comparison because it is characterized by severe genuine memory deficits and the differentiation between dementia and memory malingering is difficult for some established measured of memory malingering (Teichner & Wagner 2004;Greve et al. 2009b). On the other hand claimants in the clinical study present most often with affective disorders and genuine affective disorder patients tend to be more comparable in age to claimants than dementia patients. Moreover, affective disorder patients also often complain about their poor memory and memory deficits, although milder than in dementia, have been extensively researched in this population (Castaneda et al. 2008;McDermott & Ebmeier 2009;Mann-Wrobel et al. 2011). As in previous studies (Constantinou et al. 2005;Lange et al. 2010), probable group membership for the claimants was determined by the TOMM. An inherent methodological problem of this first study is the uncertainty of true group membership (Sollman & Berry 2011). Therefore, a second study was performed with an experimental simulation design. Non-clinical participants demographically matched to the claimants from study 1 were randomly assigned to a ‘malingering’ versus a ‘non-malingering’ group. This approach has a high internal validity (Sollman & Berry 2011).

Malingering was hypothesized to be characterized by an increase of false negative responses during immediate face recognition memory. This will result in a lower discrimination index. Inpatients with genuine organic memory deficits (i.e. dementia) should also exhibit poor overall discrimination, but this should be primarily due to a high rate of false positives. Inpatients with affective disorders might show mild memory deficits (Castaneda et al. 2008;McDermott & Ebmeier 2009;Mann-Wrobel et al. 2011), but are not expected to show as many false negatives as probable malingerers. Claimants’ not malingering and experimental controls should show a better discrimination index because they should neither show an increase of false negative responses nor an increase of false positive responses.

## Clinical study

### Methods

#### Participants

A group of 51 claimants seeking compensation was investigated. Furthermore, 51 inpatients with affective disorder and 13 inpatients with dementia were examined. Data was retrospectively obtained from all patients consecutively examined between April 2009 and December 2011, who underwent neuropsychological evaluation. The study did not require specific approval by an ethics committee and was conducted in compliance with regulations of the Department of Psychiatry and Psychotherapy of the Charité University Hospital, Berlin, Germany. Research was conducted in accordance with the Helsinki Declaration (http://www.wma.net/en/30publications/10policies/b3/index.html). Demographic information for the participants in the two groups is given in Table 1. Additional file 1: Tables S1 and S2 detail per group the distribution of ICD-10/DSM-IV diagnoses.

**Table 1:** **Neuropsychological and demographic results for the four groups of the clinical sample and the two groups of the experimental sample**

	Probably malingering claimants	Probably non-malingering claimants	Inpatients with dementia	Inpatients with affective disorder			Instructed malingering participants	Instructed non-malingering participants		
	(n = 11)	(n = 40)	(n = 13)	(n = 51)			(n = 20)	(n = 20)		
Sex (female/male)	6/5	15/25	6/7	28/23	*LR*- *χ* ^2^ = 2.97, *df* = 3, nonsignificant		12/8	10/10	*LR*- *χ* ^2^ = 0.41, *df* = 1, nonsignificant	
Analyses of Variance					*F*(3,111)	η^2^	Independent *t*-Tests		*t*-value	*df*
Age (years)	48.64^a^	45.10^a^	72.00^b^	44.00^a^	26.52***	0.42	43.70	44.20	−0.13	38
	(8.87)	(9.34)	(8.48)	(11.84)			(11.58)	(12.88)		
Years of education	11.64^ab^	13.52^a^	10.00^b^	11.55^ab^	5.00**	0.12	14.15	13.85	+0.25	38
	(2.62)	(4.03)	(3.11)	(3.28)			(3.80)	(3.73)		
Analyses of Variance					*F*(2,99)	η^2^	Independent *t*-Tests		*t*-value	*df*
WMS-LM	20.70	26.51	-	25.41	2.35	0.05	15.90	28.75	-6.44***	38
Immediate recall_a_	(6.38)	(6.61)		(8.39)			(6.82)	(5.75)		
WMS-LM	13.20^a^	20.85^ab^	-	21.31^b^	3.49*	0.07	9.95	24.35	-7.23***	38
Delayed recall_a_	(8.35)	(8.11)		(9.77)			(5.19)	(7.24)		

Note: * = *p* ≤ 0.05, ** = *p* ≤ 0.01, *** = *p* ≤ 0.001. Standard deviations appear in parentheses below means; means in the same row sharing the same superscript letter do not differ significantly from one another at *p* ≤ 0.05; means that do not share superscripts differ at *p* ≤ 0.05 based on Scheffé test post-hoc paired comparisons.

^a^WMS-LM: subtest Logical Memory of the German adaptation of the Wechsler Memory Scale-Revised.

Claimants were referred for psychiatric expert opinion by occupational disability insurance companies or from courts dealing with welfare and disability compensation issues. All claimants claimed to have cognitive deficits due to a mental disorder, requiring additional neuropsychological assessment. Full psychiatric assessment was available for 46 claimants. The most frequently reported ICD-10F/DSM-IV diagnosis was depression (46%).

Inpatients with affective disorder were routinely neuropsychologically evaluated for their cognitive performance. All received a diagnosis of an affective disorder, most of them a current depressive episode (84%). Most common comorbidities were anxiety disorders (16%), drug related disorders (12%) and psychotic disorders (12%).

Inpatients with dementia had been neuropsychologically evaluated for suspected dementia and had all subsequently received a dementia diagnosis (see Additional file 1: Table S1 and S2). Diagnoses were based on a comprehensive psychiatric and neuropsychological investigation including structural MRI findings, blood and liquor data as well as medical history from a third party. For brevity, only the Mini Mental State Examination scores (MMSE Folstein et al. 1975) are reported. The median MMSE score was 22 (*M* = 21.07; *SD* = 6.02), indicating a mild to moderate dementia.

The group of claimants was further sub-divided according to their results in the TOMM, which was administered with a discontinuation rule, as recently proposed (O’Bryant et al. 2007, 2008). If a participant scored ≥48 in trial 1 of the TOMM, the test was terminated since claimants scoring 45 or higher on trial 1 have been shown to continue to do on subsequent trials (O’Bryant et al. 2007, 2008;Gavett et al. 2005). According to the TOMM manual, claimants seeking compensation were classified as probably malingering if their test score on trial 2 was below 45 (n = 11), otherwise they were classified as probably non-malingering (n = 40).

#### Procedure and measures

For the two groups of claimants, the TOMM was administered as part of a larger neuropsychological test battery. All claimants also completed a yes/no recognition test (Alsterdorfer Faces Test, Bengner et al. 2006;Bengner & Malina 2010). This test was originally developed and validated to assess memory deficits in neurological patients. The test consists of a learning phase during which 20 unfamiliar faces are consecutively presented on a computer screen for 5 seconds each. The learning phase was followed by an immediate recognition test during which the 20 studied faces are randomly mixed with 20 new distracter faces. Participants have to decide for each face whether it was on the study list or not (yes/no). The number of hits, false positive and false negative responses and the discrimination index (P(r) = Hits - False Alarms (Snodgrass & Corwin 1988) were used as dependent variables.

Verbal memory was tested by the subtest “Logical Memory” of the German version of the Wechsler Memory Scale-Revised (Härting et al. 2000). Furthermore, 45 of the claimants filled in the German version of the Minnesota Personality Inventory-2 (MMPI 2 Hathaway et al. 2000). Here, we focus on the validity scales of this inventory. These validity scores can shed further light on the claimants’ tendency to exaggerate their symptoms (Greve et al. 2006). The Infrequency Scale (F-Scale Hathaway et al. 2000) and the Response Bias Scale (RBS Gervais et al. 2007) can be used as variables indicative of symptom exaggeration. The F-Scale contains response options which are rarely chosen by healthy controls and psychiatric patients. The RBS has been developed to discriminate between people passing and failing symptom validity tests (Gervais et al. 2007).

The same test battery, including the yes/no recognition test, but except for the TOMM and the MMPI-2, was also administered to inpatients with affective disorder.

The group of inpatients with dementia was also tested with the immediate recognition trial of the yes/no recognition test (Bengner et al. 2006;Bengner & Malina 2010). Due to the higher age of inpatients with dementia, they were otherwise examined with the German version of the Neuropsychological Assessment Battery of the Consortium for Establishing a Registry for Alzheimer Disease (CERAD-NAB Aebi 2002) which is well-standardized for this age group.

#### Statistical analyses

Univariate analyses of variance followed up by additional post-hoc Scheffé tests were used to examine differences between the two claimants groups and the two inpatient groups. Eta-squared (η^2^) was calculated to describe overall effect sizes in the ANOVA. η^2^ = 0.01 describes a small, η^2^ = 0.06 a medium and η^2^ = 0.14 a large effect (Cohen 1988). For pair-wise comparisons, effect sizes were estimated using Cohen’s *d*; *d* = 0.2 describes a small, *d* = 0.5 a medium, and *d* = 0.8 a large effect size (Cohen 1988). For ordinally scaled variables, and variables that were not normally distributed, Kruskal-Wallis Tests and Wilcoxon’s signed-rank tests were computed for group comparisons. Likelihood-Ratio *χ*^2^ tests were calculated to compare the distribution of nominally scaled variables. Phi coefficients were computed to assess the relationship between the prediction of memory malingering by the TOMM, the yes/no recognition test and the prediction of malingering by the MMPI-2 validity measures. For comparing these correlations, Steiger’s Z-test for correlated correlations within a sample was performed (Meng et al. 1992). For comparisons of multiple correlation coefficients the significance level was Bonferroni-corrected, dividing by the number of comparisons. Statistical analyses were calculated using SPSS, Version 20.0 (SPSS Inc., http://www.spss.com). Group membership for probably malingering claimants and probably non-malingering claimants was determined based on the TOMM results. Sensitivity (SN) of the yes/no recognition test was estimated on the basis of TOMM results by dividing the number of truly predicted malingerers by the base rate (BR) of malingering derived from the TOMM test (11 out of 51, i.e. 22%). Specificity (SP) was estimated by dividing the number of falsely predicted malingerers by the remaining cases (RC; 78%). The positive predictive value (PPV) and the negative predictive value (NPV) were calculated following O’Bryant and Lucas (O’Bryant & Lucas 2006): PPV = (SN × BR)/(SN × BR) + [(1 – SP) × RC] and NPV = (SP × RC)/(SP × RC) + [(1 – SN) × BR].

### Results

#### Between group comparisons

Tables 1 and 2 detail the between group comparison results. The groups did not differ in gender distribution, but inpatients with dementia were significantly older than the three other groups, who did not differ. Also, probably non-malingering claimants had significantly more years of education than inpatients with dementia, and were somewhat better educated than inpatients with affective disorder (*p* = 0.05; see Table 1).

**Table 2:** **Comparisons of the TOMM and the yes/no recognition test (Alsterdorfer Faces Test) variables between the four groups of the clinical sample**

Variable	Probably malingering claimants	Probably non-malingering claimants	Inpatients with dementia	Inpatients with affective disorder		
	(n = 11)	(n = 40)	(n = 13)	(n = 51)		
**TOMM**					*W +* value	*Z*-value
Wilcoxon signed-rank tests						
TOMM trial 1	*M* = 33.36, *S* = 8.35	*M* = 46.53, *S* = 3.95	-	-	88***	−4.56
	*Min* = 21, *Max* = 47	*Min* = 32, *Max* = 50				
TOMM trial 2	*M* = 34.55, *S* = 8.38	*M*_*a*_ = 49.53, *S*_*a*_ = 1.09	-	-	66***	−5.27
	*Min* = 16, *Max* = 43	*Min* = 45, *Max* = 50				
**Yes/No Recognition Test**					*F*(3,111)	η^2^
Analyses of Variance						
Discrimination Index P(r)	0.35^a^	0.69^b^	0.41^a^	0.68^b^	16.92***	0.31
	(0.25)	(0.21)	(0.19)	(0.15)		
False negative responses _b_	9.09^a^	3.78^b^	4.69^b^	3.47^b^	11.58***	0.24
	(4.04)	(3.16)	(2.59)	(2.55)		
False positive responses _b_	4.00^a^	2.48^a^	6.92^b^	2.94^a^	11.52***	0.24
	(3.03)	(2.01)	(3.52)	(2.34)		

*** = *p* ≤ 0.001. Standard deviations appear in parentheses below means; means in the same row sharing the same superscript letter do not differ significantly from one another at *p* ≤ 0.05; means that do not share subscripts differ at *p* ≤ 0.05 based on Scheffé test post-hoc paired comparisons.

_a_ This value is based on 26 claimants that actually performed trial 2, in 14 claimants with values ≥ 48 in trial 1, trial 2 was estimated to be 50/50.

_b_ False negative and false positive responses did not exhibit standard normal distribution measured by the Shapiro-Wilk test of normality. Parametric results are reported for readability. Results were confirmed by Kruskal-Wallis tests and post-hoc Wilcoxon´s signed-rank tests.

The discrimination index was significantly lower in the probably malingering claimants and inpatients with dementia than in the probably non-malingering claimants and inpatients with affective disorder (see Table 2^a^). Importantly, probably malingering claimants had significantly higher rates of false negative responses than the other three groups (see Table 2). Inpatients with dementia, by contrast, showed significantly higher rates of false positives than the other three groups. However, whereas the probably malingering claimants had a similar overall discrimination index as the inpatients with dementia, the groups differed in the contributing factors, namely false negatives on the one hand and false positives on the other.

Thus, probably malingering claimants and inpatients with dementia appeared quantitatively equally impaired in their face recognition memory, but they showed a qualitatively quite different pattern of responses.

#### Sensitivity & specificity

The yes/no recognition test variables were compared in their respective classification rate for predicting group membership of probably malingering claimants. A preliminary cut-off for clinical use was determined by optimizing the prediction of the receiver operator characteristic curve. False negative responses yield the best classification accuracy. A cut-off of more than 9 out of 20 possible false negative responses discriminated probably malingering claimants from probably non-malingering claimants with a sensitivity of 54% and a specificity of 95%. This cut-off had a positive predictive value of 76% and a negative predictive value of 88%. Importantly, applying this cut-off in the inpatients sample, no inpatient with dementia or affective disorder would be misclassified as a malingerer.

#### Correlations with the MMPI-2

For the 45 claimants who had completed the MMPI, prediction of malingering from the TOMM and the yes/no recognition test was correlated with prediction of elevated scores of two MMPI-2 feigning indicators. There was no significant relationship of the TOMM with the MMPI F-Scale (φ = 0.27; *p* = 0.17; *d* = 0.55^b^), or with the MMPI RBS (φ = 0.25; *p* = 0.13; *d* = 0.51). The yes/no recognition test correlated significantly with the F-Infrequency Scale (φ =0.54; *p* < 0.001; *d* = 1.27), as well as with the RBS (φ = 0.55; *p* < 0.001; *d* = 1.32). Therefore, correlations differed significantly between the TOMM and the yes/no recognition test on the F-Scale (*Z* = −2.22; *p* < 0.025; *d* = 0.69) and on the RBS (*Z* = −2.54; *p* < 0.025; *d* = 0.78).

### Study 1-discussion

In this first study, probably malingering claimants and inpatients with dementia did not differ quantitatively in their face recognition memory as measured by the discrimination index. However, in accordance with the hypothesis, they exhibited qualitatively distinct response patterns. Probably malingering claimants had an increased number of false negative responses compared with probable non-malingerers whereas inpatients with dementia had an increased number of false positive responses. Moreover, inpatients with affective disorders, comparable in age and often also reporting similar symptoms as the claimants, did not show elevated false negatives. Results suggest that yes/no recognition may be useful for discriminating between probable malingering and non-malingering, but notably also between memory malingering and genuine memory deficits on the one hand and memory malingering and affective disorder on the other.

An increased number of false positive responses in the group of inpatients with dementia (compare Table 2) is in agreement with earlier reports stating that genuine memory deficits are related to more false positive responses (Bartlett et al. 1989;Fahlander et al. 2002;Stavitsky et al. 2006;Bengner et al. 2006;Huh et al. 2006;Hudon et al. 2009;Werheid et al. 2010;Deason et al. 2012). Still, the number of false positive responses did not help to distinguish the two groups of claimants (see Table 2). The number of false negative responses, by contrast, differentiated between probably malingering and non-malingering claimants as well as probable malingerers and the two patient groups. The higher number of false negative responses of probably malingering claimants supports the hypothesis that malingerers mainly follow the naive idea that memory deficits during a recognition memory task are reflected in a total encoding failure resulting in false negative responses.

Importantly, the false negative response pattern distinguished malingerers from inpatients with dementia, and inpatients with affective disorder. The determined cut-off for false negative responses can be regarded as relatively conservative, as it did not misclassify any of the patients as a malingerer of memory deficits. This underscores the potential of false negative responses as a measure of memory malingering. By comparison, for the widely used TOMM misclassification rates for patients with moderate to severe dementia have been reported to range between 45 and 76 percent (Teichner & Wagner 2004;Greve et al. 2009b). As the TOMM was not administered to both groups of inpatients, we cannot exclude that there would have been some false positive cases of inpatients with dementia or inpatients with affective disorder in the present sample.

Moreover, there was a significant statistical relationship between the prediction of malingering from the yes/no recognition test with exaggerated scores on the MMPI-2F-Scale and RBS. As the RBS was developed to distinguish between participants passing and failing performance symptom validity tests (Gervais et al. 2007), this can be regarded as convergent validity. In the present sample, this relationship was higher for the yes/no recognition tests than for the TOMM.

Whereas no single test can replace comprehensive evaluation (e. g. MND criteria Slick et al. 1999), efficient screening methods to identify potential malingerers in a given cognitive domain are needed. On the basis of the first study, elevated false negatives could be useful in this regard. Still, a limitation of the present study is that group membership of claimants was based solely on one relatively specific measure of malingering (Vallabhajosula & van Gorp 2001;Batt et al. 2008). However, performance of the thus identified malingerers may not be representative of strategies used during malingering in general. Fortunately, in the field of malingering, unlike in other areas of clinical assessment, group status can be experimentally assigned in a simulation study (Singhal et al. 2009;Ortega et al. 2012). Therefore, in order to further establish the usefulness of a false negative responses based measure for the identification of memory malingering, a second experimental study was performed with a simulation study design.

## Experimental study

In the second study, we sought to confirm the findings of the clinical study. In an experimental design, it can be assumed that the discrepant instructions given to participants about how to respond during the investigation rather than apriori between-group differences account for different results on symptom validity and memory tests (Sollman & Berry 2011). Therefore, non-clinical participants were randomly assigned to a ‘malingering’ versus a ‘non-malingering’ group and their performance on the TOMM and the yes/no recognition test were assessed and compared. It was hypothesized that malingering would be related to a lower discrimination index largely due to an increase of false negative responses during immediate recognition memory. In the clinical study, we had specified a preliminary, rather conservative cut-off score for false negative responses as a marker of malingering. In the second study we sought to verify if using this cut-off, yes/no recognition would still have comparable classification accuracy as the TOMM.

### Methods

#### Participants

Forty volunteers were recruited via flyers at the University of Konstanz and the City of Konstanz. Participants gave written informed consent and received 10 Euros each for participation. The study did not require specific approval by an ethics committee. It was conducted in compliance with regulations at the University of Konstanz and with the Helsinki Declaration. The sample was selected to be comparable in age, education, and sex to the sample of claimants seeking compensation in the clinical study (see above and Table 3). There were no differences in age (*F(3,87)* = 0.56; *p* = 0.64), years of education (*F(3,87)* = 1.17; *p* = 0.33) or sex (Likelihood Ratio *χ*^2^ = 3.15; *df* = 3; *p* = 0.41). Demographic information for the participants of the experimental study is given in Table 1.

**Table 3:** **Comparisons of the TOMM and the yes/no recognition test (Alsterdorfer Faces Test) variables between participants instructed to malinger and participants instructed to perform normally**

Variable	Instructed malingering participants (n = 20)	Instructed non-malingering participants (n = 20)			
**TOMM**				*W +* value	*Z*-value
Wilcoxon signed-rank tests					
TOMM trial 1	*M* = 30.95, *S* = 9.73	*M* = 48.45, *S* = 2.48		218.5***	−5.25
	*Min* = 10, *Max* = 47	*Min* = 42, *Max* = 50			
TOMM trial 2	*M* = 32.95, *S* = 11.32	*M* = 49.95, *S* = 0.22		222***	−5.44
	*Min* = 6, *Max* = 50	*Min* = 49, *Max* = 50			
**Yes/No Recognition Test**			*t*-value	*df*	Cohen’s *d*
Independent t-tests					
Discrimination Index (Pr)	0.17	0.76	−7.86***	27.99	−2.49
	(0.30)	(0.15)			
False negative responses	9.65	2.85	8.05***	38	2.54
	(3.00)	(2.30)			
False positive responses _a_	6.95	1.90	4.36***	26.02	1.38
	(4.74)	(2.08)			

*** = *p* ≤ 0.001. Standard deviations appear in parentheses below means. _a_ False positive responses did not exhibit standard normal distribution measured by the Shapiro-Wilk test of normality. Parametric results are reported for readability. Results were confirmed by the Wilcoxon’s signed-rank test.

#### Procedure and measures

Participants were assigned to either a “malingering” or a “non-malingering” condition. The first 20 participants were randomly assigned. For the last 20 participants, adaptive assignment was used in order to achieve comparable demographic characteristics in both groups. Participants received instructions to either malinger cognitive deficits due to depression (n = 20) or to show full effort (n = 20). Depression was chosen because in the sample of claimants depression was the most often reported ICD-10 F/DSM-IV diagnosis and cognitive deficits due to depression were the most common complaint.

After answering a questionnaire on demographic variables, participants were instructed to pretend cognitive deficits or to show full effort (see Additional file 1 section A for the original and translated instructions). All participants completed the TOMM (Tombaugh 1996;Tombaugh 1997), the yes/no recognition test (Alsterdorfer Faces Test Bengner et al. 2006;Bengner & Malina 2010) and the subtest “Logical Memory” of the German version of the Wechsler Memory Scale-Revised (compare 4.1.2 Härting et al. 2000). Finally, all participants responded to a follow-up questionnaire about the testing procedure, which was used to validate that participants adhered to the respective instructions and which to examine whether they suspected any of the tests to be a malingering test, and if so, which one. The original and translated follow-up questionnaire is described in the Additional file 1 section B.

#### Statistical analyses

The same statistical methods as in study 1 (see 4.1.3.) were used. Malingering was experimentally assigned to half of the participants, yielding a base rate of malingering of 50%. For the yes/no recognition test the cut-off score of false negative responses >9 determined in the clinical study was used for statistical classification.

### Results

#### Between group comparisons

Statistical results are detailed in Table 3. Malingerers achieved significantly lower scores than non-malingerers on trial 1 and trial 2 of the TOMM. Addressing the main hypotheses of this study, the group of malingerers showed a significantly lower discrimination index than non-malingerers (see Table 3). This was due to both significantly higher numbers of false negative responses and significantly higher numbers of false positive responses. Still, effect size for group differences in false negative responses were larger than for false positive responses (Cohen’s *d* = 2.54 versus 1.38; see Table 3).

#### Sensitivity & specificity

The sensitivity and specificity of the yes/no recognition test and the TOMM were compared. 16 malingerers failed the cut-off score of the TOMM and 14 failed the cut-off score of the yes/no recognition test derived from study 1, while no non-malingerer failed these cut-off scores. The TOMM achieved a sensitivity of 80% and a specificity of 100%. This leads to a positive predictive value of 100% and a negative predictive value of 83%. The yes/no recognition test achieved a sensitivity of 70% and a specificity of 100%. This yields a positive predictive value of 100% and a negative predictive value of 77%. If both tests were combined and malingering was predicted if the cut-off was exceeded in one of these measures, then a sensitivity of 90% and a specificity of 100% results. This leads to a positive predictive value of 100% and a negative predictive value of 91%.

#### Questionnaire

The post-test questionnaire asked whether participants suspected any of the tests to be a malingering test, and if yes, which one. Of the instructed malingerers, eight named the TOMM. One malingerer suspected that all tests were intended to detect poor effort. No instructed malingerer singled out only the yes/no recognition test. Of the non-malingerers, three named the TOMM and one the yes/no recognition test.

### Study 2-discussion

In the experimental study, instructed malingerers revealed a higher rate of false negative responses than instructed non-malingerers during immediate yes/no face recognition. What is more, using the cut-off score for false negative responses determined in the clinical study resulted in comparable sensitivity in the detection of malingering as test results on trial 2 of the TOMM (75% versus 80%, respectively; given 100% specificity). Combining both measures, sensitivity of detecting malingering increased to 90%, given 100% specificity. Further, participants in the malingering group more often suspected the TOMM to be a test of memory malingering than the yes/no recognition test.

Instructed malingerers tend to over-exaggerate memory deficits (Greve et al. 2008), and may use different strategies than compensation-seeking claimants. Thus, the experimental study may exaggerate the true sensitivity of both measures. However, we aimed to compare sensitivity and specificity of the TOMM and the yes/no recognition test in the same experimental situation. Both methods performed comparably. Unexpectedly, the malingering group also revealed a significantly higher rate of false positive responses than the non-malingering group. This is in contrast to the result of the clinical study, and may be due to the mentioned tendency to over-exaggerate memory deficits in simulation studies leading to more disorganized behavior than in assessment situations (Greve et al. 2008). Still, the effect size for the comparison of false negative and false positive responses between instructed malingerers and non-malingerers was considerable bigger for the false negative responses.

One interesting finding from study 1 was that the yes/no recognition test was able to discriminate between truly severely memory-disordered patients, inpatients with undisputed affective disorder, and probably malingering claimants. However, a further important issue is the differentiation between experimentally instructed true malingerers and other patient groups to avoid misclassification of true disorders as malingering. Therefore, to further generalize the observed difference in false negatives between malingering and true memory disorder in study 1, experimentally instructed malingerers and the two groups of inpatients were compared on this measure.

### Between study comparisons

Compared to the two groups of inpatients, experimentally assigned true malingerers showed an even lower discrimination index (*F(2/81)* = 47.68; *p* < 0.001). This was due to the raised number of false negative responses (*F*(2/81) = 38.78; *p* < 0.001) compared to the two groups of inpatients (*p*´s < 0.001). The number false positive responses (*F*(2/81) = 15.25; *p* < 0.001) was comparable between true malingerers and inpatients with dementia (*p* = 1.0), and for both groups higher than for inpatients with affective disorders (*p*´s < 0.01). Importantly, if a false prediction of malingering for inpatients with dementia is to be avoided, no malingerer can be identified as such based on the false positive responses and only 7 malingerers can be identified by the discrimination index, while 14 malingerers can be identified by the number of false negative responses.

## General discussion

The combined results of the clinical and experimental study suggest yes/no face recognition as a useful screening tool for the detection of feigned memory deficits in claimants presenting with mental disorders. Probably malingering claimants showed a considerable increase of false negative responses in the yes/no recognition test compared to probably non-malingering claimants, but notably also compared to inpatients with dementia and established affective disorder. Although inpatients with dementia and probable malingerers showed comparable discrimination accuracy on the yes/no recognition test, these groups differed qualitatively in their response patterns. Using an analogous simulation design in the experimental study, the number of false negative responses during face recognition was found to be as good a measure of neurocognitive malingering as the TOMM. Effect sizes for differences on false negative responses were considerably large in both studies.

The results of the present paper confirm and further specify earlier observations of an unusually high number of false negative responses during the delayed yes/no recognition trial of the California Verbal Learning Test (CVLT Millis et al. 1995;Greve et al. 2009a). Whereas the CVLT is a longer and more complex memory test containing various measures, the Alsterdorfer Faces Test is a short stand-alone yes/no recognition test of immediate recognition memory. This test presents neutral faces for learning and later randomly inter-mixes the old faces with an equal number of new stimuli. While face recognition may be advantageous for the present purposes, as old targets and new distracters are structurally quite homogenous, in principle any stand-alone yes/no immediate recognition test using an immediate recognition trial may reveal a conspicuously high number of false negatives in malingerers and thus help to distinguish between malingerers and non-malingerers.

A recent meta-analysis recommends that multiple indicators of malingering should be used to achieve more accurate assessment (Sollman & Berry 2011) and clearly no single screening measure can replace comprehensive evaluation. Still, the present study suggests that the combination of a yes/no recognition test with the TOMM leads to a high sensitivity and specificity for detecting memory malingering. The presently used Alsterdorfer Faces Test is a yes/no recognition test that takes only a few minutes to conduct and score. In the clinical context, such time-saving procedures are advantageous. What is more, the Alsterdorfer Faces Test, which was originally developed to assess organic memory deficits, also contains normative data to quantify true memory deficits of claimants, if there is no evidence of malingering (Bengner & Malina 2010). Many symptom validity tests making use of the floor effect are not able to do so.

The finding that yes/no recognition test based classification results are correlated with failing MMPI-based deception scores, and more so than the TOMM-based classification, further underscores the clinical potential of the present measure.

Symptom validity tests may vary in how transparent they are to the examinee. Here, the yes/no recognition test compares favorably. In the experimental simulation sample, 8 of 20 instructed malingerers suspected the TOMM as a malingering test. In contrast, only one suspected the yes/no recognition test. This would make it more difficult for malingerers to adapt their strategy to this type of test. Finally, while the TOMM concentrates on a quantitatively conspicuous low discriminative ability, yes/no recognition tests allow focusing on the underlying qualitative response pattern.

There are limitations to the present findings. Most importantly, the sample size of the probable malingerers was relatively small. Therefore, to avoid overpowered findings in the second study, the experimental sample was also moderate. Across studies, results were quite consistent but still merit replication in larger clinical and experimental samples with diverse actual or simulated disorders. Further, classification of memory malingering in study one was based solely on the TOMM. While the TOMM is one of the most specific measures of poor effort (Vallabhajosula & van Gorp 2001;Batt et al. 2008), the administration of other indicators of malingering would be desirable. Also, claimants reported cognitive deficits due to a mental disorder and accordingly, experimental malingers were instructed to do the same. In order to generalize the present results across other potential malingering situations further studies are needed examining claimants with supposed neurological disorders using stand-alone yes/no recognition tests. The increase in false negatives should be based on a naïve idea about human memory, and therefore may be found in other cultures as well. Therefore, replications using the preliminary cut-off score in other countries would be very interesting.

## Conclusion

The present results in claimants with mental disorders are promising with regard to the detection of neurocognitive malingering, using a yes/no recognition test. In study one, probable malingerers where characterized by a selective increase of false negative responses. In the experimental study, instructed malingerers showed both more false positive and false negative responses than the non-malingers. Comparison with the performance pattern in organic deficits further underscores the utility of the false negative measure which performed well in this situation. Differentiation from patients with dementia and affective disorders reduces the problem of false positive malingering categorization in patients with moderate to severe memory disorders. Moreover, the yes/no recognition test seem to be hard to identify as a malingering detection test.

## Endnotes

^a^Analyses of covariance (ANCOVA) were calculated with age, education or both as covariates. Results were highly similar. As ANCOVAs may also be problematic in its interpretation (Miller & Chapman 2001), we report only analyses of variance.

^b^Due to multiple comparisons, the type one error is set to α < 0.025 according to Bonferroni correction for all of the following computations.

## Electronic supplementary material

Additional file 1: Table S1: Frequency of different ICD-10F diagnoses in the four groups of the clinical sample. **Table S2.** Frequency of different DSM-IV diagnoses in the four groups of the clinical sample. **A)** Instructions. **B)** Follow-up questionnaire. (DOC 90 KB)
