# Supplementary material for: Using the yes/no recognition response pattern to detect memory malingering
Source: BMC Psychol. 2013 Jun 25;1(1):12. doi: 10.1186/2050-7283-1-12 (PMC4270012; doi:10.1186/2050-7283-1-12)
Supplement: Supplementary file 1 — Additional file 1: Table S1: Frequency of different ICD-10F diagnoses in the four groups of the clinical sample. Table S2. Frequency of different DSM-IV diagnoses in the four groups of the clinical sample. A) Instructions. B) Follow-up questionnaire. (DOC 90 KB) [file 40359_2012_9_MOESM1_ESM.doc]

**Additional file**

**Table S**1: Frequency of different ICD-10 F diagnoses in the four groups of the clinical sample.

| ICD-10 F diagnoses | **Probably malingering claimants (n=9)** | | **Probably non-malingering claimants (n=37)** | **Inpatients with dementia**  **(n = 13)** | **Inpatients with affective disorder**  **(n = 51)** |
| --- | --- | --- | --- | --- | --- |
| **ICD-10 F0 diagnoses (Organic, including symptomatic, mental disorders**) F00 (Dementia in Alzheimer´s disease)  F01 (Vascular dementia)  F03 (Dementia in other diseases classified elsewhere)  F07 (personality and behavioural disorders due to brain diseases, damage and dysfunction)  G31.8* (Lewy-body dementia) | |  | **1 (3%)**  -  -  -  1 (3%)  - | **13 (100%)**  5 (38%)  4 (31%)  3 (23%)  -  1 (8%) |  |
| **ICD-10 F 1 diagnoses (Mental and behavioural disorders due to psychoactive substance use)**  F10 (Mental and behavioural disorders due to use of alcohol)  F13 (Mental and behavioural disorders due to use of sedatives or hypnotics)  F17(Mental and behavioural disorders due to use of tobacco) | |  | **3 (8%)**  2 (5%)  -  2 (5%) | **2 (15%)**  1 (8%)  -  1 (8%) | **6(12%)**  6 (12%)  2(4%)  - |
| **ICD-10 F 2 diagnoses (Schizophrenia, schizotypal and delusional disorders)**  F22 (Persistent delusional disorders)  F23 (Acute and transient psychotic disorders)  F25 (Schizoaffective disorders) | | **2 (22%)**  2 (22%)  **-**  **-** |  | **1 (8%)**  -  1 (8%)  - | **6 (12%)**  3(6%)  1(2%)  2(4%) |
| **ICD-10 F 3 diagnoses (Mood [affective] disorders)**  F31 (Bipolar affective disorder)  F32 (Depressive episode)  F33 (Recurrent depressive disorder) F34 (Persistent mood [affective] disorder) | | **4 (44%)**  -  1 (11%)  2 (22%)  1 (11%) | **17 (46%)**  -  11 (30%)  7 (19%)  4 (11%) | **6 (46%)**  1 (8%)  1 (8%)  3 (23%)  1 (8%) | **51 (100%)**  2(4%)  46(90%)  8(16%)  3(6%) |
| **ICD-10 F 4 diagnoses (Neurotic, stress-related and somatoform disorders)**  F40 (Phobic anxiety disorders)  F41 (Other anxiety disorders)  F42 (Obsessive-compulsive disorder)  F43 (Reaction to sever stress, and adjustment disorders)  F45 (Dissociative [conversion] disorders)  F48 (Other neurotic disorders) | | **7 (78%)**  3 (33%)  1 (11%)  -  4 (44%)  2 (22%)  - | **17 (46%)**  3 (08%)  5 (14%)  1 (03%)  10 (27%)  2 (05%)  2 (05%) |  | **8(16%)**  4(8%)  1(2%)  1(2%)  2(4%)  1(2%)  - |
| **ICD-10 F 5 diagnoses (Behavioural syndromes associated with physiological disturbances and physical factors)**  F50 (Eating disorders)  F55 (Abuse of non-dependence-producing substances) | | **1 (11%)**  -  1 (11%) | **1 (03%)**  1 (03%)  - |  |  |
| **ICD-10 F 6 diagnoses (Disorders of adult personality and behavior)**  F60 (Specific personality disorders)  F61 (Mixed and other personality disorders)  F62 (Enduring personality changes, not attributed to brain damage and diseases)  F63 (Habit and impulsive disorders) | | **1 (11%)**  -  1 (11%)  -  - | **10 (27%)**  5 (14%)  4 (11%)  1 (3%)  1 (3%) |  | **4 (8%)**  2(4%)  -  -  2 (4%) |
| Multiple ICD-10 F diagnoses | | 5 (56%) | 16 (43%) | 8 (62%) | 31 (61%) |

Note: Percentage of patients appears in parentheses behind the total numbers. Due to multiple F Diagnoses numbers in subgroups can be larger than the total number of F diagnoses of the respective group. For 2 probably malingering claimants and 3 probably non-malingering claimants ICD-10 F diagnoses were not yet available.

*Lewy-body dementia while not an F0 diagnoses is included there for reasons of readability.

**Table S**2: Frequency of different DSM-IV diagnoses in the four groups of the clinical sample.

| DSM IV diagnoses | **Probably malingering claimants (n=9)** | | **Probably non-malingering claimants (n=37)** | **Inpatients with dementia**  **(n = 13)** | **Inpatients with affective disorder**  **(n = 51)** |
| --- | --- | --- | --- | --- | --- |
| *Delirium, dementia, and amnestic and other cognitive disorders* 294.1 (Dementia in Alzheimer´s disease)  290.4 (Vascular dementia)  294.8 (Dementia in other diseases classified elsewhere)  310.1 (personality and behavioural disorders due to brain diseases, damage and dysfunction) | |  | **1 (3%)**  -  -  -  1 (3%) | **13 (100%)**  5 (38%)  4 (31%)  4 (31%)  - |  |
| **Substance related disorders**  305.0 (Mental and behavioural disorders due to use of alcohol)  305.4 (Mental and behavioural disorders due to use of sedatives or hypnotics)  305.1 (Mental and behavioural disorders due to use of tobacco)  305.9 (Abuse of non-dependence-producing substances) | | **1 (11%)**  -  -  -  1 (11%) | **3 (8%)**  2 (5%)  -  2 (5%)  - | **2 (15%)**  1 (8%)  -  1 (8%)  - | **6(12%)**  6 (12%)  2(4%)  -  - |
| **Schizophrenia and other psychotic disorders**  297.1 (Persistent delusional disorders)  298.9 (Acute and transient psychotic disorders)  295.7 (Schizoaffective disorders) | | **2 (22%)**  2 (22%)  **-**  **-** |  | **1 (8%)**  -  1 (8%)  - | **6 (12%)**  3(6%)  1(2%)  2(4%) |
| **Mood disorders**  296.8 (Bipolar affective disorder)  296.2 (Depressive episode)  296.3 (Recurrent depressive disorder) 300.4 (Persistent mood [affective] disorder) | | **4 (44%)**  -  1 (11%)  2 (22%)  1 (11%) | **17 (46%)**  -  11 (30%)  7 (19%)  4 (11%) | **6 (46%)**  1 (8%)  1 (8%)  3 (23%)  1 (8%) | **51 (100%)**  2(4%)  43(84%)  8(16%)  3(6%) |
| **Anxiety disorders, somatoform disorders & dissociative disorders**  300.29 (Phobic anxiety disorders)  300.0 (Other anxiety disorders)  300.3 (Obsessive-compulsive disorder)  308.3 (Reaction to sever stress, and adjustment disorders)  300.11 (Dissociative [conversion] disorders)  300.82 (Other neurotic disorders) | | **7 (78%)**  3 (33%)  1 (11%)  -  4 (44%)  2 (22%)  - | **17 (46%)**  3 (08%)  5 (14%)  1 (03%)  10 (27%)  2 (05%)  2 (05%) |  | **8(16%)**  4(8%)  1(2%)  1(2%)  2(4%)  1(2%)  - |
| **Eating disorders**  307.5 (Eating disorders) | |  | **1 (03%)**  1 (03%)  - |  |  |
| **Personality disorders**  301.x(Specific personality disorders)  301.x (Mixed and other personality disorders)  301.x (Enduring personality changes, not attributed to brain damage and diseases)  301.x (Habit and impulsive disorders) | | **1 (11%)**  -  1 (11%)  -  - | **10 (27%)**  5 (14%)  4 (11%)  1 (3%)  1 (3%) |  | **4 (8%)**  2(4%)  -  -  2 (4%) |
| Multiple DSM-IV diagnoses | | 5 (56%) | 16 (43%) | 8 (62%) | 31 (61%) |

Note: Percentage of patients appears in parentheses behind the total numbers. Due to multiple diagnoses, numbers in subgroups can be larger than the total number of diagnoses of the respective group. For 2 probably malingering claimants and 3 probably non-malingering claimants diagnoses were not yet available.

A) Instructions

*Instruction for the malingering participants:*

“Imagine you had suffered from depression, some time ago. This disease is **no longer** present. You now have no adverse effects or symptoms, anymore, and you are completely healthy. Imagine then, you would have completed a disability insurance several years ago. This insurance offers you a monthly disability pension which is only slightly lower than your regular income. Also, suppose you have claimed financial compensation from your disability insurance because of your previous episode of depression. As a reason, you had indicated that the disease leads you to exhaustion, fatigue, impaired concentration and memory problems and your ability to work is reduced by **more than half**. However, in fact, this is **not** the case, anymore. Your insurance is in doubt whether you are eligible to receive a pension, and therefore you will be examined by medical and psychological experts. So now you are to be investigated in order to determine whether such impairments really exist. To receive your financial compensation you have to pretend that you have a deficit of your memory and your concentration. Still, you also have to ensure that these simulated impairments remain credible and do not seem exaggerated. If your attempt of deception was in fact discovered, you would receive **no pension**. This would lead to serious financial problems for you.”

## ***Instruction for the non-malingering participants:***

“Imagine you had suffered from depression, some time ago. This disease led you to exhaustion, fatigue, impaired concentration and memory problems, and thus reduced your ability to work by more than half. This disease is **no longer** present. You now have no adverse effects, anymore, and you are completely healthy. You will now be examined to determine whether you still suffer from neuropsychological impairments. Your task is to demonstrate that your skills are normal, and that no impairments can be found anymore. You demonstrate this by doing the best you can in the tests that follow.”

*German original instructions for the malingering participants:*

„Stellen Sie sich vor, Sie hätten vor einiger Zeit z. B. unter einer Depression gelitten. Diese Erkrankung liegt bei Ihnen aktuell aber **nicht** mehr vor. Sie weisen nun keinerlei Beeinträchtigungen auf und Sie sind völlig gesund. Stellen Sie sich weiterhin vor, Sie hätten vor mehreren Jahren eine Berufsunfähigkeitsversicherung abgeschlossen. Diese Versicherung bietet Ihnen bei Berufsunfähigkeit eine monatliche Rente, die nicht wesentlich unter Ihrem normalen Einkommen liegt. Stellen Sie sich außerdem vor, Sie hätten nun bei dieser Versicherung aufgrund der früheren Depression vor einiger Zeit einen Antrag auf Berufsunfähigkeit gestellt. Als Begründung hätten Sie angegeben, dass die Erkrankung bei Ihnen zu Erschöpfung, Müdigkeit, Konzentrationsstörungen und Gedächtnisproblemen führt und Ihre Berufsfähigkeit um **mehr als die Hälfte** reduziert ist. Tatsächlich ist dies aber **nicht** mehr der Fall. Ihre Versicherung hat Zweifel, ob Sie berechtigt sind, eine Rente zu erhalten, und lässt Sie deshalb durch einen ärztlichen und psychologischen Gutachter untersuchen. Sie sollen also nun untersucht werden und dabei soll festgestellt werden, ob derartige Beeinträchtigungen bei Ihnen wirklich vorliegen. Um Ihre finanzielle Entschädigung zu erhalten müssen Sie bei dieser Untersuchung eine Beeinträchtigung Ihres Gedächtnisses und Ihrer Konzentration vortäuschen. Dabei müssen Sie allerdings auch darauf achten, dass diese vorgetäuschten Beeinträchtigungen glaubhaft bleiben und nicht übertrieben wirken. Sollte der Täuschungsversuch nämlich entdeckt werden, würden Sie **keine Rente** erhalten. Dies würde für Sie erhebliche finanzielle Probleme bedeuten und Ihre zukünftige Finanzierung in Frage stellen.“

*German original instructions for the non-malingering participants:*

„Stellen Sie sich vor, Sie hätten vor einiger Zeit unter einer Depression gelitten. Diese Erkrankung führte bei Ihnen zu Erschöpfung, Müdigkeit, Konzentrationsstörungen und Gedächtnisproblemen und verminderte somit ihre Berufsfähigkeit um mehr als die Hälfte. Diese Erkrankung liegt bei Ihnen aktuell nicht mehr vor. Sie weisen nun keinerlei Beeinträchtigungen mehr auf und Sie sind völlig gesund. Sie sollen nun neuropsychologisch untersucht werden um festzustellen ob Sie noch Beeinträchtigungen aufweisen. Ihre Aufgabe ist es nachzuweisen, dass Ihre Fähigkeiten normal sind und keinerlei Einbußen mehr festzustellen sind, indem Sie sich so gut wie möglich bei der folgenden Testung anstrengen.“

B) Follow-up questionnaire

1. Were there any particular difficulties during any of the tasks?
2. Do you think that some tests were specifically intended to capture for malingering of deficits (if so, which test(s)?)?
3. Have you tried to malinger specific deficits?
4. If you have malingered deficits, which deficits did you malinger?
5. If you have malingered deficits, how have you tried this?
6. If you have malingered deficits, on which tasks did you do this?
7. If you have malingered deficits, did you have particular difficulties on how to malinger?

*German original follow-up questionnaire:*

1. Gab es besondere Schwierigkeiten bei der Bearbeitung einzelner Aufgaben?
2. Glauben Sie, dass einzelne Tests speziell zur Erfassung für die Simulation von Defiziten bestimmt waren (wenn ja: welche(r) Test(s)?)
3. Haben Sie versucht bestimmte Defizite zu simulieren?
4. Wenn Sie Defizite simuliert haben, welche Defizite waren es?
5. Wenn Sie Defizite simuliert haben, wie haben Sie dies versucht?
6. Wenn Sie Defizite simuliert haben, bei welchen Aufgaben haben sie dies versucht?
7. Wenn Sie Defizite simuliert haben, gab es besondere Schwierigkeiten bei der Simulation von Defiziten bei einzelnen Aufgaben?
